# Comparison of CO_2_ Separation Efficiency from Flue Gases Based on Commonly Used Methods and Materials

**DOI:** 10.3390/ma15020460

**Published:** 2022-01-08

**Authors:** Zenon Ziobrowski, Adam Rotkegel

**Affiliations:** Institute of Chemical Engineering, Polish Academy of Sciences, 44-180 Gliwice, Poland; arot@iich.gliwice.pl

**Keywords:** carbon dioxide, absorption, adsorption, membrane separation

## Abstract

The comparison study of CO_2_ removal efficiency from flue gases at low pressures and temperatures is presented, based on commonly used methods and materials. Our own experimental results were compared and analyzed for different methods of CO_2_ removal from flue gases: absorption in a packed column, adsorption in a packed column and membrane separation on polymeric and ceramic membranes, as well as on the developed supported ionic liquid membranes (SILMs). The efficiency and competitiveness comparison of the investigated methods showed that SILMs obtained by coating of the polydimethylsiloxane (PDMS) membrane with 1-ethyl-3-methylimidazolium acetate ([Emim][Ac]) exhibit a high ideal CO_2_/N_2_ selectivity of 152, permeability of 2400 barrer and long term stability. Inexpensive and selective SILMs were prepared applying commercial membranes. Under similar experimental conditions, the absorption in aqueous Monoethanolamine (MEA) solutions is much faster than in ionic liquids (ILs), but gas and liquid flow rates in packed column sprayed with IL are limited due to the much higher viscosity and lower diffusion coefficient of IL. For CO_2_ adsorption on activated carbons impregnated with amine or IL, only a small improvement in the adsorption properties was achieved. The experimental research was compared with the literature data to find a feasible solution based on commercially available methods and materials.

## 1. Introduction

The observed growth of greenhouse gas emissions, mainly from fossil fuels combustion in industry, have stimulated the development of new technologies for CO_2_ removal and storage [1,2].

European Union Emission Trading Scheme (EU ETS) is the biggest strategy to control greenhouse gas emissions with its carbon tax and carbon allowances [3,4]. In 2019, the carbon tax level was about 100 €/ton of CO_2_ equivalent and the carbon allowance price level was above 30 €/ton of CO_2_ equivalent [5]. Within the EU ETS control policy, greenhouse gas emissions should be 41% lower in 2030 than in 2005. The price of CO_2_ emission allowances is rising and reached 60 €/ton CO_2_ in 2021, even though it was predicted to reach 40 €/ton CO_2_ by 2023 [6].

In order to regulate these emissions, carbon capture and storage (CCS) and carbon capture, utilization and storage (CCUS) techniques have been widely used [7,8]. The carbon dioxide capture and separation is the first step of these techniques and its cost is estimated to be as much as 80% of the total CCS cost [9,10]. An important part of CCUS is the carbon dioxide utilization step, which is regarded as the most challenging and has potential to reduce the world’s current annual CO_2_ emissions by 10% [11].

The main approaches for CO_2_ capture are pre-combustion, post-combustion and oxy-combustion processes [12,13]. In post-combustion processes, CO_2_ concentration in flue gas is about 10 to 15% vol., pressure is near atmospheric, and the temperature is usually in the range of 313–348 K [14]. The CO_2_ concentrations and pressures are higher for CO_2_ separation from natural gas [15].

Generally, the following methods have been used for carbon dioxide capture from gases: absorption, adsorption, membrane separation, cryogenic distillation, hydrate-based CO_2_ capture, chemical-looping combustion and biological separation using bacteria or algae [11,16]. Among these methods, absorption, adsorption and membrane separation are regarded as the most established and mature.

### 1.1. Absorption

Currently, among the available separation methods, the amine scrubbing processes using MEA are widely used in coal-fired power plants for CO_2_ capture [17,18]. It is the most advanced technology for CO_2_ removal, and its advantage is high absorption efficiency (greater than 90%). Sorbents can be easily regenerated by heating or depressurization. The disadvantages being reported are degradation of amines with temperature and time, corrosion, amine losses by evaporation, toxicity of solvents used in absorption processes [19,20], high capital and operation costs [21], high energy requirement [22] and a high amount of heat for sorbent regeneration [23]. The sorbent regeneration for primary and tertiary amines may increase the total operating costs by up to 70% when the heat of the reaction is high [9,10].

It was reported by Ramdin et al. [24] that approximately 2.5–3.6 GJ is required to remove one ton of CO_2_ using a 30 wt.% aqueous MEA solution. Lucquiaud et al. [25], Jackson and Brodal [26] have found that the energy required for a CCS plant is about 250–300 kWh/t CO_2_, whereas the estimated energy needed for CO_2_ compression is 80–120 kWh/t CO_2_.

For a typical amine scrubbing process using MEA solution, the energy consumption is approximately 3.8 MJ/kg CO_2_, while the energy needed for regeneration is about 3.22 MJ/kg CO_2_ [27]. Li et al. [28] reported that for amine scrubbing process the minimum reboiler duty is 3.1 MJ/kg CO_2_.

Optimizing important process parameters, the height of absorber, stripper, the reboiler duty could save about 20% of the heat consumption, thus improving the efficiency of the process [29,30,31,32].

In past decades, as an alternative to traditional amines, ionic liquids and deep eutectic solvents (DESs) have been considered as potential replacements for CO_2_ capture [33,34,35,36]. 

Their properties—tunability, chemical and thermal stability, high CO_2_ solubility, negligible vapor pressure, and a more environmentally friendly character—allow them to be used as alternative CO_2_ absorbents. The literature reviews show a variety of synthesized ILs [37,38] and DESs [39,40,41] for CO_2_ capture and the need to look for a reliable screening procedure linking molecular characteristics of ILs and DESs to their overall performance in carbon capture processes.

The proper selection of individual IL components and molar ratios in the case of DESs may allow us to prepare a specific and unique solvent that is suited for a particular application. A major issue in the case of IL applications, especially in comparison with low-cost DESs, are their high viscosity and price. Most of the investigated ILs absorb CO_2_ physically. This mechanism is responsible for a low CO_2_ loading and an easier CO_2_ desorption than in the case of MEA solutions [42]. 

Compared to amine-based solvents, conventional ILs exhibit a low CO_2_ absorption capacity. The CO_2_ solubility in post-combustion processes is less than 5% mol., as a result of low partial pressure of CO_2_ at post-combustion conditions [43]. In order to increase the CO_2_ absorption capacity in ILs, new task-specific ionic liquids (TSILs) were developed, as functionalized ILs, by introducing an amino group (NH_2_) into the IL. In functionalized ILs, as opposed to conventional ILs, CO_2_ absorption occurs by chemical reaction and the CO_2_ loading capacity is comparable to MEA solution.

In 2009, Bara et al. [44] obtained a CO_2_ loading capacity comparable to that of an MEA solution, which represents an interesting alternative to amine scrubbing processes [36,45,46,47]. Shifflet et al. [48,49] investigated the CO_2_ phase behavior in imidazolium-based ILs [Bmim][Ac] and [Emim][Ac]. They found that these ionic liquids containing acetate anion showed a strong CO_2_ absorption at pressure 2 MPa and in the temperature range of 10–75 °C. 

The presence of an amine moiety in the anion [50,51] or in anion and cation [52] of imidazolium-based ILs increased CO_2_ absorption capacities of the corresponding conventional ILs. The positioning of the amine moiety at the cation of IL or at the anion of IL enables carbamate formation with 1:2 or 1:1 reaction stoichiometry, respectively.

Shiflett et al. used [Bmim][Ac] as a CO_2_ absorbent, and performed the simulation of the CO_2_ separation process and compared it with the MEA-based scrubbing process. They reported that [Bmim][Ac] can replace an MEA solution in a coal-fired power plant (180 MW). Compared to the MEA-based scrubbing process, the energy losses were lowered by 16% and the investment costs by 11% [53]. 

It was found that using [Emim][Ac] in the process of carbon dioxide removal from flue gas, the energy requirements were lower but the investment costs were higher in comparison with the MEA-based process [54]. There are several pilot projects based on ionic liquids, yet capture data are unavailable.

### 1.2. Adsorption

Adsorption is another recommended method for CO_2_ capture from post-combustion gases because of its high adsorption capacity and efficiency, which is greater than 85%, its low capital investments, its lower regeneration energy requirements and its ease of handling. The achieved purity of CO_2_ can be higher than 95% [55,56,57]. Moreover, the process is reversible. The regeneration step may be realized by vacuum, pressure, or temperature swing adsorption (VSA, PSA, TSA) [58]. 

Many different adsorbents were investigated: zeolites, mesoporous silica, clays, metal–organic frameworks (MOFs) and activated carbons [59]. These adsorbents are not widely used for economical and technical reasons; high desorption energy and high temperature adsorbents are required.

Porous-activated carbons exhibit better adsorption than other adsorbents, their energy consumption at the regeneration step is low, and for this reason activated carbons are often used in industry [60].

A great research effort was directed to develop proper surface and pore structures as well as new functionalized activated carbons to obtain enhanced CO_2_ adsorption capacity and optimize the breakthrough time [61].

He et al. [62] investigated the dynamic of adsorption of gas containing 15% CO_2_ vol. on coconut-shell-activated carbons before and after grafting and impregnation with novel phosphonium-based IL for different gas flow rates and adsorption pressures. They found that the CO_2_ adsorption capacity of the investigated activated carbons at 0.1 MPa and 25 °C had changed from 10 to 7 wt.% after functionalization with IL, while and ideal CO_2_/N_2_ selectivity had distinctly increased from 7 to 30. 

Mesoporous silica are materials that are frequently used for adsorbent preparation because of their easily modifiable structural properties [63]: high surface area, large, tunable pore diameter volume. Silica showed a rather low CO_2_ adsorption, but the addition of amino groups to silica support allow for silica modification and development of functionalized adsorbents for CO_2_ [64]. The functionalization method is of great importance in CO_2_ adsorption. The objective of the functionalization method makes for an improvement of the adsorption capacity by introducing specific groups to the surface of the adsorbent. This can be done by a grafting technique or by a chemical impregnation technique under dry or wet conditions.

In the grafting method, the specific groups are bonded chemically through covalent bonding to the solid support. Thus, modified silica acquire more stable properties and faster kinetics because of their stronger interactions. Hiremath et al. [65] used 1-methyl-3-ethylimidazolium-based IL grafted on mesoporous silica functionalized with Lysine-IL and found a CO_2_ adsorption capacity of 0.61 mmol/g-adsorbent at 298 K. 

Zhang et al. [66] found a CO_2_ adsorption capacity of 2.15 mmol/g-adsorbent at 333 K and 0.15 bar using a wet impregnation method. They impregnated functionalized mesoporous silica SBA-15 with tetraethylenepentaammonium nitrate ([TEPA][NO_3_]). 

The impregnated silica have a greater adsorption capacity than grafted silica [67]. 

Solid adsorbents developed through amine-functionalization adsorbed CO_2_ by chemisorption and follow the carbamate formation scheme. The reported CO_2_ adsorption capacities are in the wide range from 0.1 to 5.91 mmol/g-adsorbent depending on experimental conditions and investigated materials. The typical enthalpy values for chemisorption and for physical adsorption are between 40 and 90 kJ/mol and between 15 and 40 kJ/mol, respectively [67]. This means that CO_2_ molecules are more strongly bound to the surface of amine-functionalized solid adsorbents by both chemical reactions and physical interactions with silica support [68]. For raw silica materials, physical adsorption takes place mainly via van der Waals interactions.

### 1.3. Membrane Separation

Membrane gas separation is one of the most mature and advanced methods for gas separation. It is considered as an alternative method for carbon dioxide removal in relation to the amine-based scrubbing processes. Its advantages are low energy demands, simple maintenance [69,70], an environmentally friendly character, low cost of the polymeric membranes and its variety of manufacturers [71,72]. The obstacles are low permeability and selectivity, poor stability, aging, swelling and sensitivity to the content of impurities and water [73,74]. 

Different materials (organic and inorganic) were tested for CO_2_ separation [75,76]. A commonly used cellulose acetate, polyimides, fluorinated polyimides, polyether-urethane-urea, polyether-block-amide show low permeability in the range from 2.4 to 212 and low ideal CO_2_/N_2_ selectivity (α_CO2/N2_) in the range from 12.5 to 36.7 [77,78,79,80,81,82]. New advanced polymeric materials with CO_2_ separation potential are presented and studied such as polymers with intrinsic microporosity (PIM) or thermally rearranged polymers (TR); however, their application needs more research [83]. 

It was found that common PDMS membranes provide a high permeability and can be used for carbon dioxide capture from post-combustion gases [84,85]. These membranes keep their properties and do not undergo swelling or degradation [86]. The PDMS membranes possess a very high CO_2_ permeability of 4000 barrer but a low ideal CO_2_/N_2_ selectivity of 2.6 [87].

In previous years, SILMs have been used for selective gas separation. SILMs may be developed by impregnation of the porous support with an ionic liquid. The application of ILs for CO_2_ removal averts the shortcomings of amine-based processes [88,89]. Ionic liquids have properties such a high carbon dioxide solubility, a negligible vapor pressure, and thermal stability, which allow them to be used as effective carbon dioxide absorbents. The application of ILs in carbon dioxide absorption may result in significant investment and operation cost reduction [90,91]. Unfortunately, their high viscosities and prices are their important disadvantages. 

Different membrane supports made of polymeric or inorganic materials and different ILs have been tested. Cserjési et al. [92] investigated hydrophilic polyvinylidene fluoride (PVDF) support and 12 different room temperature ionic liquid RTILs. For prepared SILMs, the measured CO_2_ permeabilities were from 94 to 750 barrer and α_CO2/N2_ from 10.9 to 52.6. Santos et al. [93] also used PVDF support and prepared SILMs by impregnating PVDF with the following ionic liquids: 1-ethyl-3-methylimidazolium acetate ([Emim][Ac]), 1-butyl-3-methylimidazolium acetate ([Bmim][Ac]) and vinylbenzyltrimethylammonium acetate ([Vbtma][Ac]). For investigated ILs in the temperature range from 25 to 60 °C, they found carbon dioxide permeability from 852 to 2114 barrer and ideal CO_2_/N_2_ selectivity from 26.4 to 39.

Bara et al. [94] studied imidazolium-based room temperature ionic liquids (RTILs) and found carbon dioxide permeability in the range from 210 to 320 barrer and ideal CO_2_/N_2_ selectivity from 16 to 26.

Albo et al. [95] used [Emim][Ac to impregnate porous Al_2_O_3_/TiO_2_ tubes. The measured CO_2_ permeability and ideal selectivity were 780 barrer and 35.4, respectively. Sánchez Fuentes et al. [96] investigated functionalized ceramic SILMs with amino group at the anionic part of IL. They obtained a high CO_2_ permeability of 3000 barrer and high α_CO2/N2_ of 70.

Khraisheh et al. [97] used microporous polysulfone matrix (PSF) impregnated with different concentrations of ionic liquids: 1-Ethyl-3-methylimidazolium hexa fluorophosphate ([Bmim][PF_6_]) and bis(trifluoromethylsulfonyl) imide ([Emim][Tf_2_N]). The small addition of IL to the PSF matrix enhanced both CO_2_ permeability and selectivity. The measured CO_2_ permeabilities were from 10.8 to 13.8 barrers and α_CO2/N2_ from 33 to 37.2. The correct impregnation is very important for the stability of the liquid phase in an SILM [98].

The goal of this work is to present and compare the competitiveness and efficiency of CO_2_ separation from flue gases based on commonly used methods and materials. Our own experimental study of CO_2_ removal from post-combustion gases is presented for different methods at low pressures (1–5 bar) and temperatures (20–60 °C). The following advanced CO_2_ capture methods were compared: absorption and adsorption in a packed column and membrane separation on ceramic and polymeric membranes, as well as on developed SILMs. 

Packed columns are the standard technical solution used in many industrial processes. However, large capital costs and the size of apparatus are limiting factors for an efficient application of this technology. The membrane processes permit the removal of these limitations. This technology is often used in industry and is considered environmentally friendly; it does not emit any gases or liquids. 

This work also presents the comparison of the separation efficiency for SILMs prepared by impregnation of the ceramic support of commercial membranes made by INOPOR and Pervatech BV with ionic ILs: [Emim][Ac], [Bmim][Ac], [Emim][Tf_2_N] and [Emim][BF_4_]. The aim of this part of the research was to determine the stability and separation enhancement for SILM developed by the addition of an IL-separating layer to commercial membranes. 

Additionally, the experimental research is compared with the literature data to find a feasible, economically reasonable and environmentally friendly solution based on commercially available methods and materials.

## 2. Experimental Results and Discussion

Experimental research is presented for the following CO_2_ capture methods: absorption, adsorption and membrane separation. The experiments were carried out on experimental setups described in detail in previous works [99,100,101]. The main parts of the experimental setups were packed columns with CO_2_ liquid solvents or solid adsorbents and a membrane separation module with ceramic and polymeric membranes, as well as the developed SILMs.

### 2.1. Absorption in a Packed Column

A 15 wt.% MEA solution [Emim] [Ac] and [Bmim] [Ac] were used as the solvents for CO_2_ absorption in a packed column sprayed with IL. [Emim] [Ac] and [Bmim] [Ac] were taken into account due to literature reports about their high absorption capacity and chemical type of absorption (chemisorption). 

For these solvents, the sorption capacity and the time of complete saturation with CO_2_ was determined using a bubble-type apparatus [99]. This apparatus consisted of a thermostatic 2 dm^3^ glass reactor, a mixer and a gas bubbler. Carbon dioxide was introduced at the bottom of the glass reactor and was absorbed in the MEA solution or ionic liquid at temperatures 20, 40, 60 °C and atmospheric pressure. 

**Figure 1:**
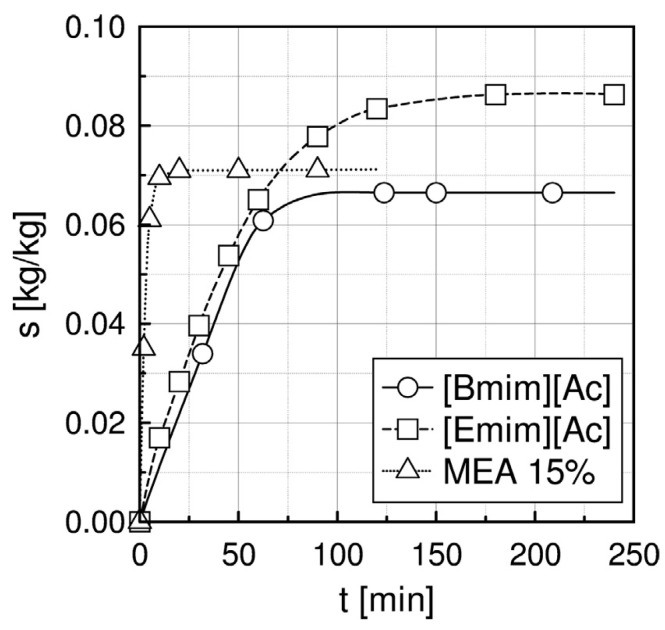
The CO_2_ sorption capacity at 1 bar, temperature 40 °C, gas flow rate V_g_ = 36 L/h.

The CO_2_ absorption capacity, S, in the investigated solvents was calculated as a ratio of the absorbed mass of CO_2_ [kg] and the mass of the solvent absorbing CO_2_ [kg]. The results are shown in Figure 1 [99]. 

At the temperature of 40 °C, atmospheric pressure and gas flow rate V_g_ = 36 L/h, the CO_2_ absorption capacities for [Bmim][Ac] and [Emim][Ac] were 0.067 and 0.086 [kg_CO2_/kg_IL_] respectively. In the same conditions, the absorption capacity for 15 wt.% MEA was close to these values, 0.071 [kg_CO2_/kg_MEA_]. Similar results were reported in the literature (0.077 and 0.079 for [Bmim][Ac] and [Emim][Ac], respectively) [45]. Additionally, it can be seen in Figure 1 that the absorption profile for MEA is steeper than for IL, which indicates that the CO_2_ absorption rate in MEA is higher than in both ILs. 

The obtained comparable results of CO_2_ absorption capacity in selected ionic liquids and MEA solutions led to an attempt to use these ionic liquids as CO_2_ absorbents in a packed column. The experimental setup described in [100] consisted of a packed glass column with an inner diameter of 0.05 m, a length of 0.35 m filled with glass Rashig rings of a diameter 5 × 1 mm and a length of 5 mm. The column was heated with a water jacket. The ionic liquid or MEA solution flowed through the bed and absorbed CO_2_ from the gas mixture in co-current or countercurrent flow. The experiments were carried out at atmospheric pressure and in temperatures of 20, 40 and 60 °C.

The CO_2_ absorption in ILs was performed in a limited range of flow rates to avoid flooding of the column. In the co-current flow (gas 1–2.3 L/min and liquid 0.05–0.2 L/min) and in the countercurrent flow (gas 1–2.1 L/min and liquid 0.05–0.1 L/min). The absorption temperature determines the efficiency of CO_2_ capture. The minimum absorption temperature was set at 40 °C because [Bmim][Ac] solidifies at 30 °C. 

The regeneration step (desorption process) was carried out at the temperature 90 °C, using pure nitrogen to help stripping CO_2_. The regenerated IL was used again. In the case of MEA, for each experiment, new 15 wt.% MEA-water solution was used. The amount of absorbed CO_2_ in the absorption process (3–4 h) and amount of desorbed CO_2_ in desorption process (6–8 h) was controlled gravimetrically until measured changes were negligible (0.1 g).

**Figure 2:**
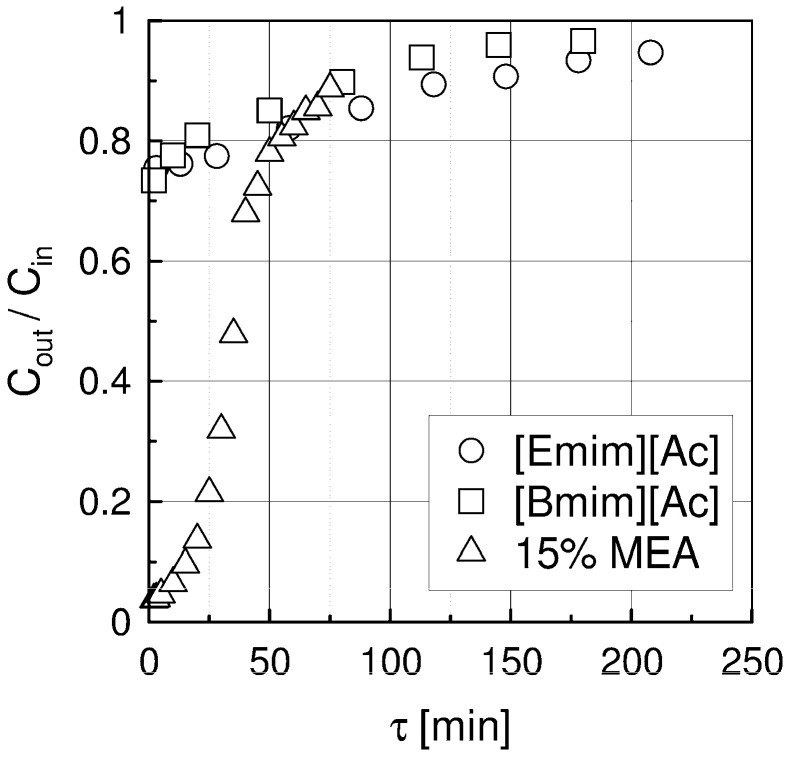
Comparison of outlet CO_2_ concentrations for [Emim][Ac], [Bmim][Ac] and 15 wt.% MEA at 1 bar, temperature 40 °C, Cin = 15% vol. and gas flow rate V_g_ = 138 L/h.

**Figure 3:**
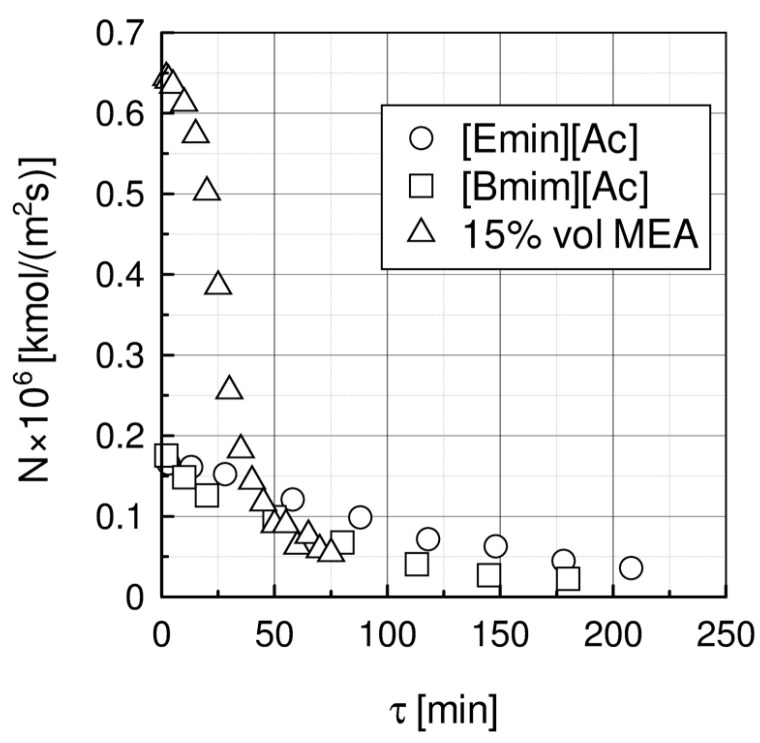
Comparison of molar fluxes of absorbed CO_2_ for investigated liquids at 1 bar, temperature 40 °C, Cin = 15% vol. and gas flow rate V_g_ = 138 L/h.

The experimental results are presented in Figure 2 and Figure 3. The comparison of carbon dioxide absorption in the investigated solvents is presented in Figure 2 for co-current flow, inlet CO_2_ concentration 15% vol. and absorption temperature of 40 °C and gas flow rate V_g_ = 36 L/h. The outlet CO_2_ concentration, Cout, after passing through the column, is low at the beginning because in the liquid phase there is no CO_2_ and almost all CO_2_ in the gas phase is absorbed. With time, the amount of CO_2_ absorbed in liquid decreases and thus the outlet CO_2_ concentration rises until complete saturation, when Cout = Cin. The experiments were carried out until Cout = (0.90–0.98) Cin. For Ils, the initial outlet CO_2_ concentration is higher and the time when concentration Cout/Cin ≥ 0.95 is longer in comparison with the 15 wt.% MEA solution. 

The measured initial outlet CO_2_ concentration Cout/Cin are: 0.035, 0.721 and 0.754 for MEA solution, [Bmim][Ac] and [Emim][Ac], respectively. The time needed to reach outlet CO_2_ concentration Cout = 15% vol. is about 80 min for MEA solution and 210 min for [Emim][Ac] and [Bmim][Ac]. 

The influence of absorption temperature and flows direction on outlet CO_2_ concentration is of minor effect.

In Figure 3, the measured CO_2_ molar fluxes are compared. The initial CO_2_ molar flux for 15 wt.% MEA solution is significantly higher and decreases faster with time than for ILs. 

Experimental results show that imidazolium-based ionic liquids can be applied in a packed column for CO_2_ removal from post-combustion gases. 

In Table 1, physical parameters of the CO_2_ absorption in a packed column are presented for: temperature 40 °C, co-current flow, inlet CO_2_ concentration 15% vol.

The viscosities of both ILs are very high and as a consequence, mass transfer coefficients in the liquid phase are much lower than for 15 wt.% MEA.

Comparison of mass transfer coefficients in liquid and gas phase shows that the CO_2_ absorption process in a packed column is controlled by a liquid side mass transfer resistance. The liquid side mass transfer coefficient, as well as the initial CO_2_ molar flux for [Bmim][Ac] and [Emim][Ac] are several times lower than for 15 wt.% MEA solution. Absorption capacities, S, are comparable for all of the investigated liquids.

### 2.2. Adsorption in a Packed Column

The adsorptive CO_2_ removal research was carried out on an experimental setup equipped with a stainless steel column of diameter 50 mm and length 800 mm. The column was thermostated by Thermostat Lauda Eco Gold with accuracy ±0.2 °C. The following beds were investigated: molecular sieves type 4A (4 mm) made by Chempur, pelletized activated carbon (4 mm) made by Elbar-Katowice Sp z o.o. and pelletized activated coconut carbon (4 mm) impregnated with triethylenediamine (TEDA)—PHS 4S TEDA made by Eurocarb Products Limited and granulated activated coconut carbon (2 mm). The height of the investigated beds was about 700 mm. 

To measure CO_2_ concentrations, a gas chromatograph Varian Star 3800 and PORAPLOT Q 25 m long megabore column and TCD detector was used for GC component analysis with accuracy ±0.01%.

Before measurements, the bed was heated in an electric oven at the temperature of 120 °C for 24 h. The prepared CO_2_/N_2_ gas mixture with CO_2_ concentration in the range of 3–12% vol. was preheated to the column temperature and introduced at the bottom of the column. Flow meter and rotameters with accuracy ±20 mL/min were used to measure gas flow rates. Pure gases CO_2_ and N_2_ (purity 99.99%) were used to prepare an inlet gas mixture. At the top and bottom of the column and gas inlet/outlet, NiCr-Ni thermocouples with accuracy ±0.2 °C were used to measure and control the temperatures of the bed.

The inlet/outlet CO_2_ concentrations were measured with time to determine the amount of CO_2_ adsorbed in the bed. Additionally, the bed was weighed before and after the experiments to control concentration measurements.

To improve the CO_2_ removal, the beds were later impregnated with ionic liquid [Emim] [Ac]. The impregnation was made by soaking the previously heated bed in 50 wt.% IL-isopropanol solution for 24 h. Thus, the prepared bed was dried and heated for the next 48 h and then used in experiments at an atmospheric pressure.

**Figure 4:**
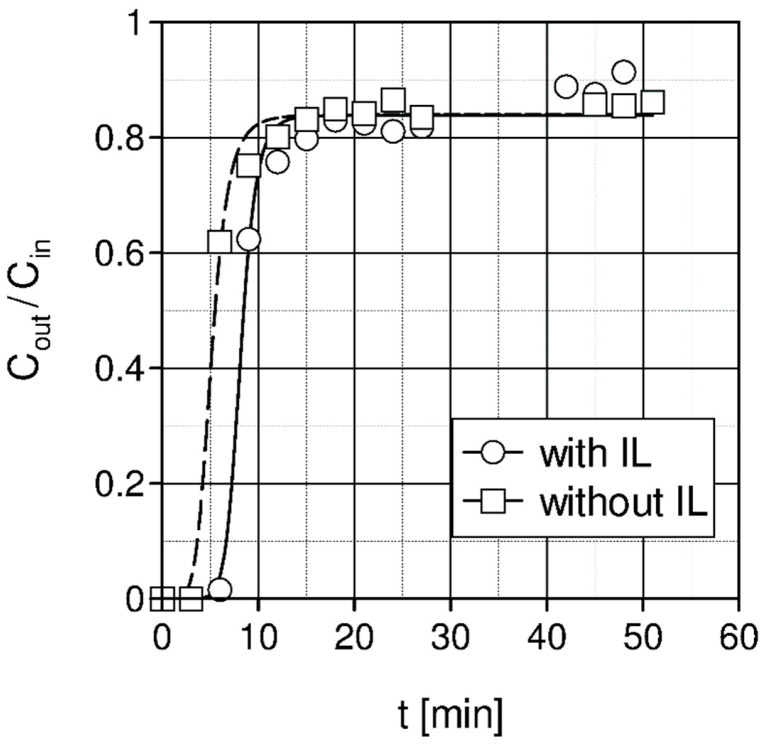
Comparison of measured CO_2_ concentration for adsorption on pelletized activated carbon (4 mm) bed before and after its impregnation with ionic liquid [Emin][Ac] (1 bar, temperature 20 °C, Cin = 10% vol. and gas flow rate Vg = 750 L/h).

**Figure 5:**
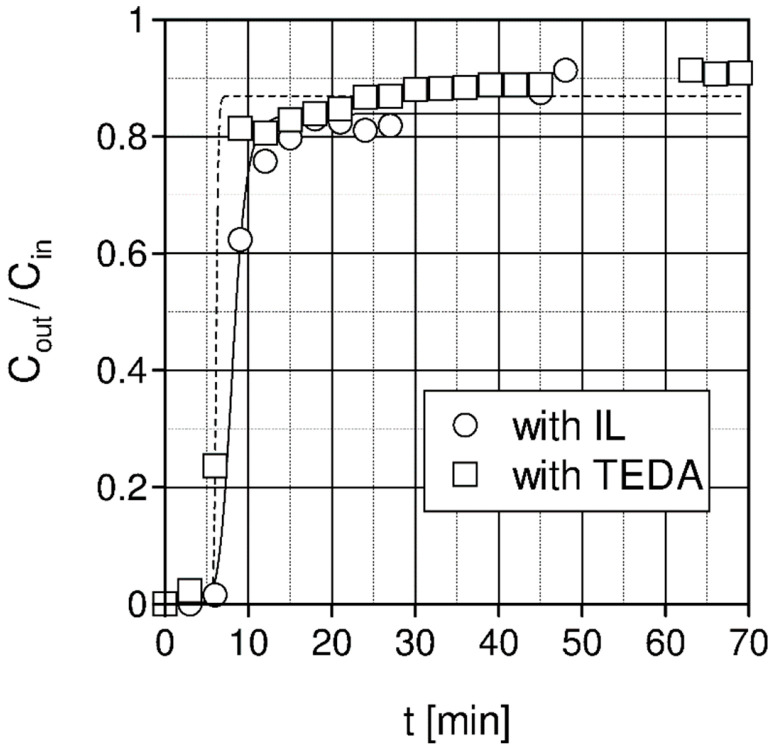
Comparison of measured CO_2_ concentration for adsorption on pelletized activated carbon (4 mm) bed impregnated with ionic liquid [Emin][Ac] and on pelletized activated carbon coconut (4 mm) bed impregnated with TEDA amine (1 bar, temperature 20 °C, Cin = 10% vol. and gas flow rate Vg = 750 L/h).

The experimental results are presented in Figure 4 and Figure 5 for pelletized activated carbon (4 mm) made by Elbar-Katowice Sp z o.o. and pelletized activated coconut carbon (4 mm) impregnated with TEDA made by Eurocarb Products Limited for the temperature of 20 °C and Cin = 10% vol.

In Figure 4, pelletized activated carbon (4 mm) impregnated with ionic liquid [Emim][Ac] has a slightly higher sorption capacity than pelletized activated carbon (4 mm) without impregnation. Furthermore, breakthrough of the column bed occurs later in the case of pelletized activated carbon (4 mm) impregnated with IL. If it is assumed that the column breakthrough occurs when the outlet concentration reaches 10% of the inlet concentration value, then for the column packed with activated carbon without IL, the breakthrough time is 4.6 min, and for the same column impregnated with IL, the breakthrough time was increased to 7.6 min. Unfortunately, the improvement of CO_2_ absorption capacity and capability of CO_2_ removal is not satisfactory.

Figure 5 presents a comparison of the measured outlet CO_2_ concentration for adsorption of the inlet gas containing 10% vol. of carbon dioxide (Cin = 10% vol.) on pelletized activated carbon (4 mm) impregnated with IL [Emin][Ac] and on pelletized-activated coconut carbon (4 mm) impregnated with amine TEDA. The CO_2_ sorption capacity results obtained in both beds are similar and are equal to S = 0.013 [kg_CO2_/kg_adsobent_] for pelletized activated carbon (4 mm) impregnated with [Emin][Ac] and S = 0.012 [kg_CO2_/kg_adsobent_] for pelletized activated carbon (4 mm) impregnated with TEDA. He et. al. [62] reported adsorption capacity obtained in a fixed bed column (7 mm inner diameter and 150 mm in height) for activated carbon impregnated with phosphonium ionic liquid at 2 atm and 25 °C equal 0.029 [kg_CO2_/kg_adsobent_]. The column breakthrough time defined above is similar and was 5.3 and 7.6 min for TEDA and [Emin][Ac], respectively. 

The shape of the column breakthrough curve indicates that in the case of TEDA, the adsorption capacity of the bed is slightly lower, while the diffusion rate is much higher than in the case of an ionic liquid. 

Research carried out on activated carbon impregnated with ionic liquids showed a slight increase in the sorption capacity of the modified adsorbents, as well as a slight increase in their CO_2_ adsorption properties. This may be due to the blockage of adsorbent pores by a viscous ionic liquid.

The regeneration of the bed was carried out in an electric oven at the temperature of 120 °C for 24 h. 

### 2.3. Membrane Separation

The membrane separation research was carried out on the experimental setup, described in detail in our previous work [101]. The main part of the setup is a stainless steel module with a tubular ceramic membrane. The following commercial tubular ceramic membranes with outer diameter of 0.1 m, an inner diameter of 0.007 m, and a length 0.25 m were used in this research:

A—membranes made by Inopor with active TiO_2_ layer and pore diameters 10, 30, 100 nm;

B—membranes made by Inopor with active Al_2_O_3_ layer and pore diameters 5, 10 nm (γ-Al_2_O_3_) and 70 nm (α-Al_2_O_3_);

C—membranes made by Pervatech with active PDMS layer and pore diameter of ceramic support 100 nm.

Ionic liquids were purchased from Sigma-Aldrich: [Emim][Ac] (97.8%), [Bmim][Ac] (95%), [Emim][Tf_2_N] (95%), [Emim][BF_4_] (97%). Before the experiments, ILs were purified by vacuum for about 24 h. 

In Table 2, some physical and thermal ILs properties are presented in standard conditions.

Significant differences in solubility values between presented ILs can be attributed to a different absorption mechanism: chemisorption in case of [Emim][Ac] and [Bmim][Ac] or physisorption in case of [Emim][BF_4_] and [Emim][Tf_2_N]. To immobilize IL in the pores of the ceramic membrane support, two impregnation methods were applied: coating and soaking. 

The mass of IL added to the membrane was controlled by weighing of the membrane before and after impregnation. Similarly, before and after each series of experiments, the membrane was weighted to control its mass or weight loss.

Ideal CO_2_/N_2_ selectivity was calculated according to Equation (1).

(1) $$\alpha_{{\mathrm{CO}}_{2}/N_{2}}=\frac{P_{{\mathrm{CO}}_{2}}}{P_{N_{2}}}$$

where *P_i_* is the permeability of component *i* [kmol m−^1^ s^−1^ Pa^−1^], *P_i_* is a product of diffusivity (*D_i_*) and solubility (*s_i_*) of the component *i.*

Molar flux (*N_i_*) [kmol m^−2^ s^−1^] for gas *i* was calculated according to Equation (2):

(2) $$N_{i}=\frac{D_{i}s_{i}}{l}\Delta p=\frac{P_{i}}{l}\Delta p$$

where *l* is the membrane thickness [m], Δ*p_i_* is the pressure difference [Pa].

CO_2_ and N_2_ gases of purity 99.99% were used. When the membrane module was prepared and ready for experiments (residual gases were removed under vacuum, the required temperature was achieved), the pressure of feed gas was increased up to 500 kPa with 50 kPa steps. A Varian Digital flow meter was used to measure gas flow through the membrane. The measurements were repeated for both feed gases: N_2_ and CO_2_. 

**Figure 6:**
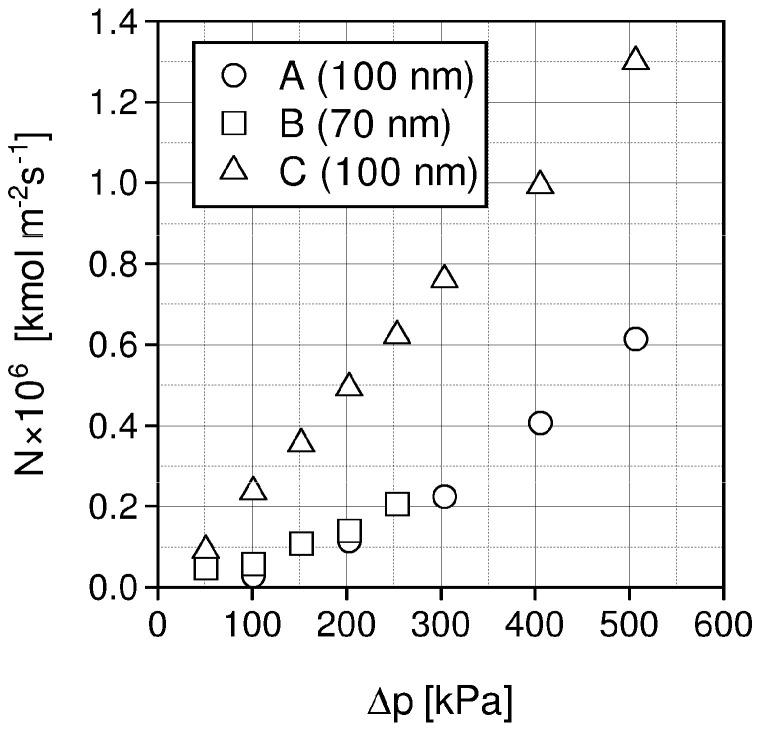
Comparison of CO_2_ molar fluxes for SILMs based on membranes A, B and C impregnated with [Emim][Ac], temperature 20 °C.

**Figure 7:**
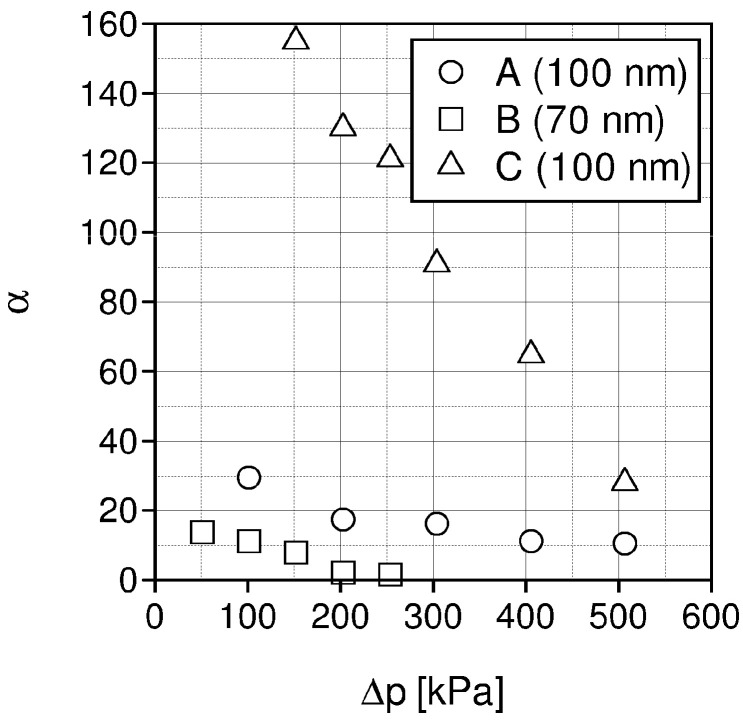
Ideal CO_2_/N_2_ selectivity for SILMs based on membranes A, B and C impregnated with [Emim][Ac], temperature 20 °C.

**Figure 8:**
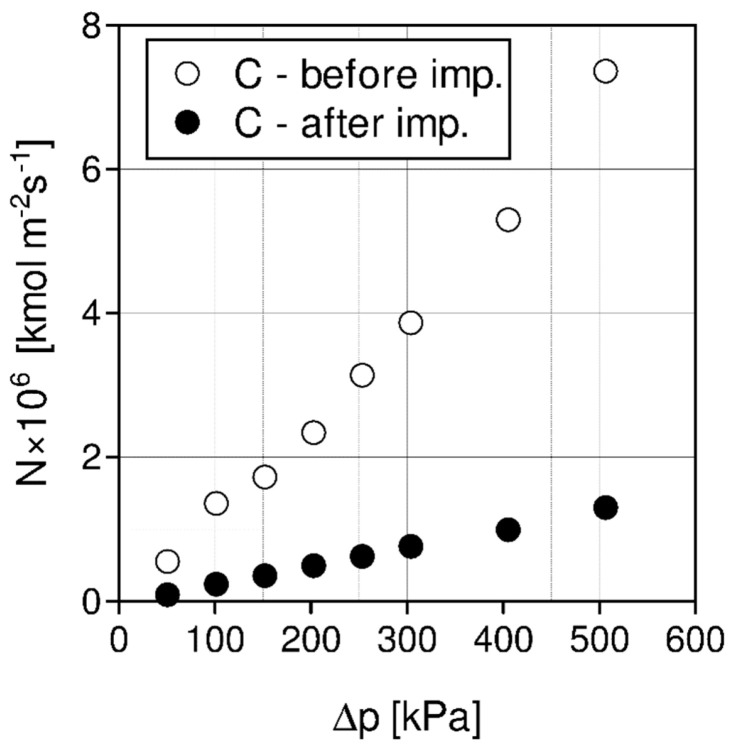
Carbon dioxide molar fluxes for membrane C (PDMS) before and after impregnation with [Emin][Ac], temperature 20 °C.

**Figure 9:**
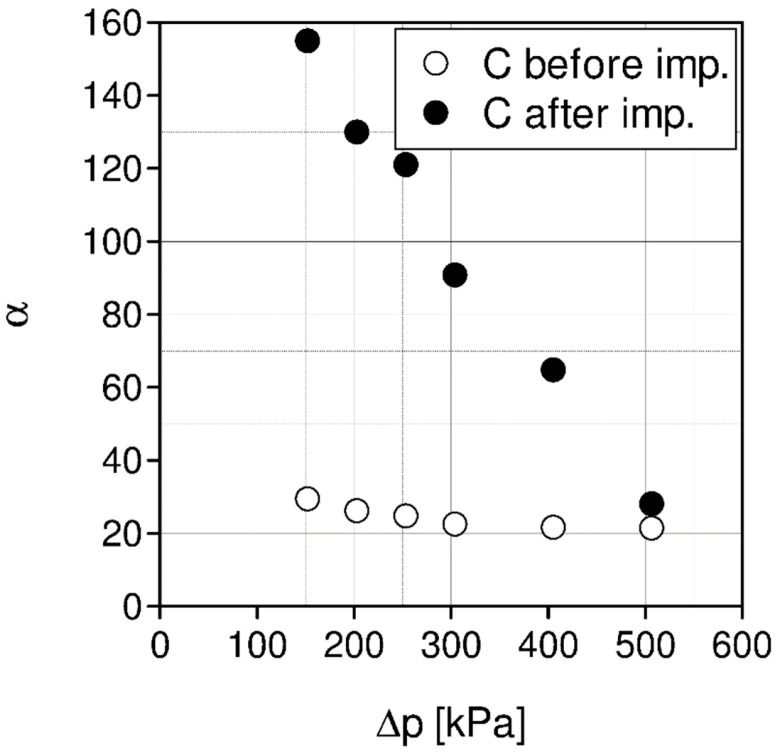
Ideal CO_2_/N_2_ selectivities for membrane C (PDMS) before and after impregnation with [Emin][Ac], temperature 20 °C.

Based on the chosen membranes A, B, and C, the SILMs were developed by impregnation with different ILs: [Emim][Ac], [Emim][Tf_2_N], [Emim][BF_4_] by coating and soaking methods. The effects of pressure, temperature, pore diameter and impregnation method on CO_2_/N_2_ separation were investigated [101]. Some experimental separation results are presented in Figure 6, Figure 7, Figure 8 and Figure 9. 

In Figure 6, carbon dioxide molar fluxes are presented for the developed SILMs, prepared by coating of the membranes A, B, C with [Emim][Ac].

The measured CO_2_ molar fluxes, for membrane C (PDMS) after impregnation, are distinctly greater than molar fluxes for membranes A and B in the same experimental conditions. Nitrogen molar fluxes were very small and were not presented in Figure 6. The differences between CO_2_/N_2_ molar fluxes may be explained by a permeation mechanism, which for N_2_ is controlled by diffusivity, but for CO_2_ is controlled by CO_2_ solubility in IL. With the rising pressure difference, the driving force is rising and thus the measured CO_2_ molar fluxes also increase.

As can be seen in Figure 7, with the rising pressure, the ideal CO_2_/N_2_ selectivities decrease. In the case of the SILMs prepared by impregnating the membrane C (PDMS}, high selectivities were obtained.

The greatest measured ideal CO_2_/N_2_ selectivities for SILMs based on membrane A and B are equal 30 and 15, respectively, and are significantly lower than for membrane C (PDMS), at about 152.

In Figure 8, carbon dioxide molar fluxes are presented for the same membrane C (PDMS) before and after impregnation with [Emim][Ac] for the temperature of 20 °C. As can be noticed, after impregnation, the CO_2_ molar fluxes are considerably lower because of additional mass transfer resistances of the IL layer. 

High ideal CO_2_/N_2_ selectivities measured for a SILM developed by coating with [Emim][Ac] of the membrane C (PDMS) can be explained by the effect of an additional layer formed by impregnating the ceramic tube with [Emim][Ac]. High selectivity is also proof that the coating method in this case is an efficient and economically reasonable way of preparing a highly selective SILM. [Emim][Ac] does not dissolve in PDMS. The impenetrable PDMS layer keeps the ionic liquid in the pores of the support, thus helping to maintain long-term stability and improving the performance of the prepared SILM. The separation mechanism may be described as resistance in a series model [93] with a CO_2_ chemical absorption in the IL layer and CO_2_ solution-diffusion in the PDMS layer. 

The measured ideal selectivities are much higher after impregnation of the membrane C (PDMS) with ionic liquid [Emim][Ac], Figure 9.

The low cost and stability of the thus prepared SILM is an interesting alternative compared to SILMs based on expensive materials and advanced functionalized ILs [83,96].

The thickness of the PDMS layer—30 μm—was given by the manufacturer. The IL layer thickness was estimated to be 210 μm for coating and 450 μm for soaking, taking into account the membrane weight after impregnation.

A proper realization of the coating process may help to achieve a better separation performance of the prepared SILMs [95].

**Figure 10:**
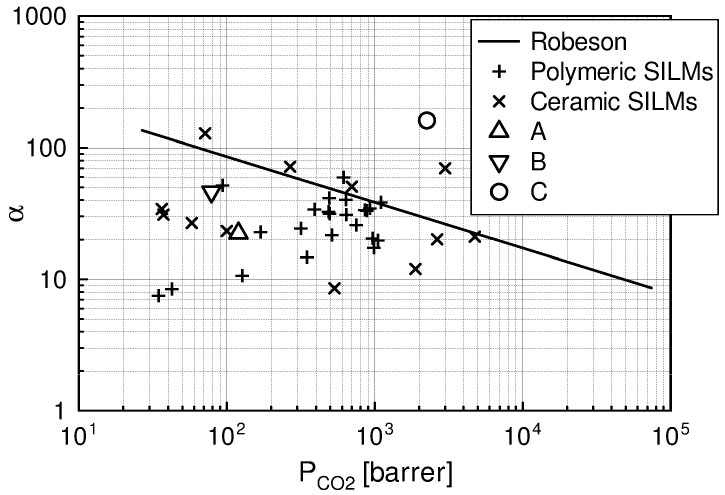
Separation performance comparison for investigated SILMs and literature data: A—Membrane A impregnated with [Emim][Ac], B—Membrane B impregnated with [Emim][BF_4_], C—Membrane C (PDMS) impregnated with [Emim][Ac].

In Figure 10, the developed SILMs were compared with the literature data for polymeric [92,93,102,103] and ceramic [95,96,104,105,106] SILMs, as well as the revised upper bound Robeson correlation (2008) [107]. The experimental results lying above this correlation are considered an improvement in separation efficiency of the investigated SILMs.

The best results were obtained for SILMs based on membrane C (PDMS) prepared by coating of the ceramic support with [Emim][Ac]: high ideal CO_2_/N_2_ selectivity of 152 and permeability of 2400 barrer. These results lie above the literature data and above the Robeson upper bound correlation.

The results for membranes A and B, impregnated by coating with [Emim][Ac] and [Emim][BF_4_], respectively, lie below the Robeson upper bound correlation. For membrane A, the ideal CO_2_/N_2_ selectivity and permeability were 24 and 140 barrer and for membrane B, 45 and 90 barrer, respectively.

The main disadvantage of the SILMs is their insufficient stability, which is important in the case of large-scale industrial applications and long-time operations [108,109]. The SILM stability depends strongly on the ILs properties and the preparation methods [110,111].

### 2.4. Comparison of Process Parameters for the Investigated Methods

The general comparison of measured CO_2_ sorption capacities and CO_2_ molar fluxes for the investigated methods of CO_2_ removal is presented in Table 3. The comparison was made for the following experimental conditions: temperature of 40 °C, atmospheric pressure, ionic liquid [Emim][Ac] as a CO_2_ solvent and inlet CO_2_ concentration 12–15% vol. Calculated CO_2_ sorption capacity S represents the mass (kg) of absorbed CO_2_ per mass (kg) of IL or the bed for absorption or adsorption, respectively, during the time of the experiment.

As can be seen in the case of [Emim][Ac] as a CO_2_ solvent, the absorption in the packed column allows for obtaining high sorption capacities and molar fluxes. For membrane separation, high selectivity α was measured, but CO_2_ molar fluxes were very low. For adsorption, the measured values were rather small but comparable with the literature data. Maximum absorption or adsorption capacity S was obtained for saturation of [Emim][Ac] with pure CO_2_ for about 8 h.

Regeneration step in the case of absorption of CO_2_ in pure IL was made by heating of IL at 95 °C under vacuum for 12 h. For absorption in a packed column, the regeneration was made “in situ” at a temperature of 90 °C with inert gas (nitrogen) flow, for 12 h. For adsorption in the packed column, the regeneration step was performed by heating of the bed in an electric oven at the temperature of 120 °C, for 24 h. In the case of membrane separation, some of the prepared SILMs lost their separation properties because of the loss of IL from the pores of the ceramic support at elevated pressures. It is possible to use the same ceramic support again for the same IL by additional impregnation with IL, but it needs a special effort to clean and prepare the membrane. Such experiments were performed, but it is easier and safer to prepare the new membrane.

## 3. Conclusions

The experimental research is presented for different methods of carbon dioxide removal from flue gases: absorption, adsorption and membrane separation under the same or similar experimental conditions, based on commonly used materials: packings, beds, membranes and CO_2_ solvents. The experiments were carried out at low pressures and temperatures for the chosen standard imidazolium ILs and the materials were modified by impregnation with IL or amine. 

The efficiency comparison of the investigated methods showed that for an SILM based on a ceramic membrane C (PDMS) impregnated with [Emim][Ac] by coating in a vacuum (Figure 10), the best results of long-term stability and permselectivity were obtained with a high value of ideal selectivity and permeability 152 and 2400 barrer, respectively. High separation coefficient values can be crucial in cases where selectivity is a priority, even when permeate molar fluxes are very low.

Applying commercial tubular ceramic membranes made by Inopor and Pervatech, inexpensive SILMs were prepared by impregnating them with selected ionic liquids by coating or soaking methods. For the prepared SILMs, CO_2_ molar fluxes increase and the ideal CO_2_/N_2_ selectivities decrease with the increasing pressure difference and feed temperature. The low cost of PDMS membranes and the small amount of ionic liquid required for impregnation, coupled with a simple method of IL immobilization in the membrane support make it possible to obtain stable and highly selective SILM membranes.

A presentation of the obtained results in Robeson’s plot shows an improvement in separation efficiency and selectivity. The separation performance of SILMs formed by ionic liquid impregnation of membranes A and B made by Inopor is below the limit given by Robeson.

The optimum operating conditions for the tested SILM membranes were a feed temperature of 20 °C and a pressure below 200 kPa. With the higher transmembrane pressures, SILMs lose permselective properties due to degradation.

For CO_2_ absorption in a packed column sprayed with IL, gas and liquid flow rates were limited due to the high viscosity of the ionic liquids. For the higher flow rates, an effect of column flooding was observed. The inlet CO_2_ concentration and temperature significantly affects the absorption efficiency.

Despite similar carbon dioxide absorption capacities, under the same experimental conditions, the absorption in aqueous MEA solution is much faster than in ionic liquids. This effect is due to the much higher viscosity of ILs rather than amine solutions, so the diffusion coefficient for ILs is lower than for amine solutions.

For CO_2_ adsorption on activated carbons, pelletized activated carbon (4 mm) and pelletized activated coconut carbon (4 mm) impregnated with ionic liquid ([Emim][Ac]) or amine (TEDA), respectively, only a small improvement in the adsorption properties was achieved.

The comparison of investigated methods (Table 3), taking into account [Emim][Ac] as a CO_2_ solvent, shows that applying a packed column sprayed with IL allows for obtaining high CO_2_ sorption capacities and molar fluxes. In the case of membrane separation, high selectivities α were measured, but CO_2_ molar fluxes were low. For adsorption on activated carbons, measured values of CO_2_ sorption capacities were rather small, but were comparable with the literature data.

The experimental research and results may represent interesting clues for a decision on which method of carbon dioxide removal will be more efficient for a specific task.

## Figures and Tables

**Table 1:** The physical parameters of the CO_2_ absorption process in a packed column.

Liquid	ρ kg/m^3^	η × 10^3^ Pa s	D_CO2_ × 10^10^ m^2^ s^−1^	k_L_ × 10^8^ kmol/(m^2^ s)	k_G_ × 10^8^ kmol/(m^2^ s)	N_exp,τ = 0_ × 10^6^ kmol/(km^2^ s)	S kg/kg	Price *^)^ €/kg
[Emim][Ac]	1.025	66.98	4.21	9.90	3.08	0.166	0.053	350
[Bmim][Ac]	1.050	145.3	2.51	6.04	3.08	0.177	0.043	460
15% MEA	0.999	0.938	22.4	204	3.08	0.668	0.049	20

*^)^ prices by Proionic https://proionic.com (accessed on 5 November 2021).

**Table 2:** Physical and thermal properties of ILs (0.1 MPa, 298.15 K).

Liquid	M kg/kmol	ρ kg/m^3^	η × 10^3^ Pa s	CO_2_ Solubility % mol.	D_CO2_ × 10^10^ m^2^ s^−1^	Price *^)^ €/kg
[Emim][Ac]	170.21	1.025	66.98	26.7	4.21	350
[Bmim][Ac]	198.26	1.050	145.3	19.4	2.51	460
[Emim][BF_4_]	197.97	1.27	34.0	2.0	5.95	620
[Emim][Tf_2_N]	391.31	1.52	32.6	3.0	5.6	690

*^)^ prices by Proionic https://proionic.com (accessed on 5 November 2021).

**Table 3:** Comparison of process parameters for the investigated methods of CO_2_ removal at 40 °C and atmospheric pressure.

Investigated Methods with [Emim][Ac]	C_in_ (CO_2_% vol.)	Gas Flow, V (l^3^/h)	P (atm)	N·10^6^ (kmol m^−2^ s^−1^)	S (kg CO_2_/kg Sorbent)/α	Regeneration Step
Absorption in pure liquid	100	36	near atmospheric	^-^	s = 0.086	thermal t = 95 °C and vacuum
Absorption in packed column	15	138	near atmospheric	0.166	s = 0.053	thermal t = 90 °C with N_2_
Adsorption in packed column	12	750	near atmospheric	0.111	s = 0.013	thermal t = 120 °C
Membrane separation	12	-	2–6	0.025–0.1	α = 10–136	n/a

## Nomenclature

barrer	non-SI unit of gas permeability; 1 barrer = 3.35 × 10^−16^ (mol m s^−1^ Pa^−1^ m^−2^)
D	membrane diffusion coefficient, m^2^ s^−1^
l	membrane thickness, m
N	molar flux, kmol m^−2^ s^−1^
P	permeability, kmol m^−1^ s^−1^ Pa^−1^
p	pressure difference, Pa
s	solubility, kmol m^−3^ Pa^−1^
S	sorption capacity, kg CO_2_ kg^−1^ sorbbent
V_g_	gas flow rate, L/h
α	ideal selectivity
ρ	density, kg m^−3^
Subscripts	
CO_2_	carbon dioxide
i	CO_2_, N_2_
in	inlet
N_2_	nitrogen
out	outlet
